# Longitudinal phenotyping of heart failure with preserved ejection fraction identifies early- and end-stage disease states

**DOI:** 10.1136/openhrt-2026-004351

**Published:** 2026-09-25

**Authors:** Samuel Brown, Fardad Soltani, Jack Wu, Matthew Ryan, Brett S Bernstein, Brian Tam To, Tom Searle, Maleeha Rizvi, Natalie Fairhurst, George Kaye, Ranu Baral, Dhanushan Vijayakumar, Daksh Mehta, Zeeshan Khawaja, Phil Chowienczyk, James Teo, Richard JB Dobson, Daniel I Bromage, Gerald Carr-White, Thomas F Lüscher, Ali Vazir, Theresa A McDonagh, Jessica Webb, Christopher A Miller, Ajay M Shah, Dhruva Biswas, Kevin O’Gallagher

**Affiliations:** 1 King’s College Hospital NHS Foundation Trust, London, UK; 2 School of Cardiovascular and Metabolic Medicine & Sciences, British Heart Foundation Centre of Research Excellence, King’s College London, London, UK; 3 Division of Cardiovascular Sciences, The University of Manchester, Manchester, UK; 4 Department of Biostatistics and Health Informatics, Institute of Psychiatry, Psychology and Neuroscience, King's College London, London, UK; 5 Guy's and St Thomas’ NHS Foundation Trust, London, UK; 6 King’s College London GKT School of Medical Education, London, UK; 7 Royal Brompton & Harefield NHS Foundation Trust, London, UK; 8 Manchester University NHS Foundation Trust, Manchester, UK

**Keywords:** Electronic Health Records, Heart Failure, Diastolic, Risk Factors

## Abstract

**Background and aims:**

Heart failure with preserved ejection fraction (HFpEF) phenotypes have been characterised as static entities in trial populations yet their temporal dynamics remain unexplored in routine clinical practice. We aimed to identify HFpEF phenogroups from real-world electronic health records (EHRs), characterise their longitudinal stability and determine whether phenogroup trajectories were associated with differences in mortality.

**Methods:**

A natural language processing (NLP) pipeline identified HFpEF cases from multisite EHRs (2010–2022). Latent class analysis (LCA) defined baseline phenogroups and annual supervised random forest (RF) classification using time-updated values of the same baseline LCA variables tracked individual transitions over five years. Competing risks regression quantified transition versus death probabilities. Time-varying Cox models assessed mortality risk associated with current (vs baseline) phenogroup status. Phenogroups were independently re-derived by LCA in an external cohort and compared with RF predictions.

**Results:**

Among 2223 patients (60% female, median age 75 years (IQR 63–83); left ventricular ejection fraction (LVEF) 60.1%; follow-up 4.0 years (IQR 2.2–6.1), we identified four phenogroups: young-low comorbidity (22%), obesity-predominant (24%), elderly atrial dysfunction (32%) and cardiovascular-kidney-metabolic (23%). Young-low comorbidity had the highest transition probability (47.7%), while cardiovascular-kidney-metabolic and elderly-atrial dysfunction were highly stable (3.2% and 12.1% transitioned, respectively). Within young-low comorbidity, those who transitioned had greater baseline cardiac abnormalities, including higher E/e′, left ventricle mass and wall thickness. Adjusted mortality risk was highest in cardiovascular-kidney-metabolic (HR 1.53, 95% CI 1.22 to 1.92, p<0.001); current phenogroup status discriminated mortality risk modestly better than baseline classification alone (C-index 0.659 vs 0.648). In an independent external cohort (n=3349), RF predictions agreed strongly with independently derived LCA phenogroup labels (C-statistics 0.891–0.953).

**Conclusion:**

Longitudinal NLP-based phenotyping of real-world EHRs identified four reproducible HFpEF phenogroups with distinct trajectories, distinguishing an early progressive disease state from stable advanced phenotypes. Earlier HFpEF detection using artificial intelligence-driven EHR tools could facilitate phenotype-targeted treatment of patients at potentially modifiable stages.

**WHAT IS ALREADY KNOWN ON THIS TOPIC**

- Heart failure with preserved ejection fraction (HFpEF) phenotyping studies have identified distinct comorbidity-defined subgroups, but these analyses have been largely cross-sectional, leaving phenogroup stability and progression over time unexplored.

**WHAT THIS STUDY ADDS**

- Serial machine learning classification of real-world electronic health record data demonstrates that HFpEF phenogroups are dynamic; a young-low comorbidity phenogroup represents an early progressive disease state, identifiable by baseline diastolic and structural abnormalities, while advanced phenogroups are stable and carry the highest mortality.

**HOW THIS STUDY MIGHT AFFECT RESEARCH, PRACTICE OR POLICY**

- Identifying an early progressive HFpEF phenotype may provide an opportunity for earlier identification and phenotype-targeted intervention in those in whom disease modification remains possible.

## Introduction

 Heart failure with preserved ejection fraction (HFpEF) accounts for approximately half of all heart failure cases worldwide with a mortality burden comparable to heart failure with reduced ejection fraction.^1 2^ Despite this, HFpEF remains underdiagnosed and undertreated in routine care.^3–5^ Our previous work demonstrated that only 8% of patients meeting European Society of Cardiology (ESC) diagnostic criteria had a documented HFpEF diagnosis,^5^ underscoring a substantial gap in real-world recognition. This under-recognition may partly reflect the marked clinical heterogeneity of HFpEF,^6^ which is a barrier to timely diagnosis and thus early intervention.

Analyses of large registry and trial cohorts have identified distinct HFpEF phenogroups characterised by differences in comorbidity burden, cardiac structure and prognosis.^7–12^ However, most prior studies examined highly selected populations with HFpEF variably defined,^7–9 12–14^ potentially limiting applicability to routine clinical practice. Critically, these analyses were cross-sectional. Whether phenogroups represent fixed disease states or dynamic stages along a common trajectory remains unknown.^15^ Understanding transitions between phenotypes could clarify HFpEF progression, distinguish early from advanced disease and identify modifiable stages amenable to intervention.

To overcome these limitations, we used an electronic health record (EHR) cohort of patients meeting ESC diagnostic criteria for HFpEF, identified using an artificial intelligence (AI)–enabled natural language processing (NLP) pipeline.^5 16–18^ Importantly, rather than characterising phenogroups at a single timepoint, we applied repeated supervised machine learning classification to time-updated clinical data, enabling longitudinal tracking of individual phenogroup transitions over five years. We sought to identify clinically distinct HFpEF phenogroups in routine clinical practice, characterise their stability over time and determine whether phenogroup trajectories were associated with differences in mortality.

## Methods

### Study population

We used our previously published HFpEF cohort, derived from EHRs at King’s College Hospital (KCH) and Guy’s and St Thomas’ (GSTT) NHS Foundation Trusts (2010–2022).^5 16 17^ This cohort represents patients meeting contemporary ESC diagnostic criteria for HFpEF,^19^ with clinical characteristics and biomarker profiles typical of HFpEF registries in the absence of alternative diagnoses. The original derivation of our cohort has been published in detail previously.^5^ Patient inclusion followed a structured, stepwise approach, requiring patients to fulfil all criteria (online supplemental figure S1):

1. Clinician-documented heart failure (≥2 mentions of ‘heart failure’ or ‘HF’ in the EHR, identified via NLP).
2. Left ventricular ejection fraction (LVEF) ≥50% within 12 months of the first HF mention. Patients were excluded if they had a recorded LVEF <50% at any time before or after the index HF mention.
3. At least one marker of diastolic dysfunction/raised left ventricle (LV) filling pressures: N-terminal pro-B-type natriuretic peptide (NT-proBNP) ≥125 pg/mL (≥ 375 pg/mL with atrial fibrillation (AF)), LV mass index ≥95 g/m^2^ (female) 115 g/m^2^ (male), left atrial (LA) volume index >34 mL/m^2^ (> 40 mL/m^2^ with AF), E/e’ ratio >9, pulmonary artery systolic pressure >35 mm Hg or tricuspid regurgitation velocity >2.8 m/s.^19^

### Data extraction

Medical Concept Annotation Tool within the CogStack platform was used to extract Systematized Nomenclature of Medicine—Clinical Terms concepts from the EHR,^20 21^ including all inpatient documentation, clinic letters, discharge summaries and referrals. In addition to identifying mentions of HF, the NLP pipeline extracted explicitly documented ‘HFpEF’ or ‘heart failure with preserved ejection fraction’, as well as comorbidities (type 2 diabetes mellitus, hypertension, chronic kidney disease (CKD), AF, myocardial infarction, coronary artery disease (CAD), cerebrovascular accident (CVA) and medications (loop diuretics, angiotensin-converting enzyme (ACE) inhibitors, angiotensin II receptor blockers, beta-blockers and calcium channel blockers (CCB)). The tool accounted for synonyms, for example, ‘HF’ and applied contextual filters to exclude negations, hypothetical mentions and terms referring to individuals other than the patient. The date of diagnosis was defined as the first HF mention. Previous manual validation of a random representative sample of 100 NLP-extracted ‘HFpEF’ mentions has demonstrated a 100% true-positive rate for correctly identifying clinician-recorded HFpEF documentation.^16^ Structured data provided demographics, body mass index (BMI), echocardiography reports, natriuretic peptides, haemoglobin, creatinine and haemoglobin A1c (HbA1c). For analysis, BNP/NT-proBNP levels (available in 70% of the cohort) were combined into a single log-transformed z-score, following established methodology.^8^ H_2_FPEF and HFpEF-ABA scores were calculated from baseline data.^22 23^ Mortality data were obtained from EHR death notification letters. As per the stepwise approach, after confirmation of a clinician-documented HF, the classification of HFpEF was determined independently using structured EHR data according to ESC criteria, irrespective of whether HFpEF was explicitly clinician-documented in clinical notes. See online supplemental method S1 for more detail.

### Clustering approach and variable selection

Phenogroups were identified using latent class analysis (LCA), an unsupervised clustering approach that applies maximum likelihood estimation to classify individuals into mutually exclusive subgroups based on shared characteristics. By identifying latent subgroups through associations between multiple clinical features rather than a single trait, LCA is particularly well suited for HFpEF phenotyping.^7 9 13 14^ Alternative methods, including hierarchical clustering and model-based approaches, generated an excessive number of subgroups, limiting their clinical applicability. A total of 10 variables were selected for clustering based on known associations with HFpEF prognosis and clustering approaches in prior studies.^7 9 13 14^ Variables included age (<65 years, 65–75 years, 75–85 years and >85 years), sex, BMI (WHO classifications: <18.5 kg/m^2^, 18.4–25kg/m^2^, 25–30kg/m^2^, >30kg/m^2^), hypertension, AF, diabetes, CKD, CAD and smoking history and natriuretic peptide quartiles. Missing baseline continuous data (online supplemental table S1) were imputed using the *missForest* package in R prior to clustering. LCA was implemented using the *poLCA* package in R,^24^ with model estimation iterated 20 times across 2–10 potential subgroups. The optimal number of phenogroups was determined using the first Bayesian Information Criterion minima to balance model fit with clinical interpretability (online supplemental table S2). Posterior class membership probabilities were generated for each individual (online supplemental table S3), representing the likelihood of belonging to each phenogroup given an observed characteristic. Patients were then assigned to the phenogroup with the highest posterior probability, corresponding to maximum-likelihood classification (online supplemental table S4). To quantify the magnitude of between-phenogroup differences in variables used to characterise the phenogroups, standardised mean differences (SMDs) were calculated for all LCA variables. SMDs were calculated for each of the six pairwise phenogroup comparisons, with the mean pairwise SMD reported for each variable.

### Longitudinal phenogroup tracking

To analyse phenogroup transitions over time, we leveraged time-stamped longitudinal data available within our NLP-extracted dataset. A supervised random forest (RF) classifier was trained on baseline LCA variables to predict phenogroup membership. The model hyperparameters were optimised to minimise out-of-bag error. At each annual timepoint from the first heart failure mention, each patient’s clinical profile was updated to include comorbidities, BMI and natriuretic peptides that appeared in the EHR up to and during that interval. New diagnoses were defined as the first mention appearing after the index HF diagnosis date. Once recorded, comorbidities were considered present at all subsequent timepoints. The RF model was then applied to these updated clinical profiles to determine phenogroup membership at each timepoint up to five years. This approach ensured that the same four baseline-defined phenogroups were used throughout follow-up, allowing changes in classification to reflect changes in an individual’s clinical profile rather than changes in the phenogroup definitions themselves. For patients with irregular follow-up schedules, we used the most recent clinical data available within ±2 months of each annual timepoint. Where no updated data were available within this window, the most recently recorded prior value or comorbidity status was carried forward. This approach assumed clinical stability in the absence of a documented change. Death was treated as a separate competing outcome, allowing us to distinguish between phenotype transitions and mortality outcomes. Phenogroup stability was assessed using patients with <10% change in LCA model variables from baseline. Model confidence was evaluated using RF-assigned probabilities, with high confidence defined as >90%. To characterise differences between patients who transitioned between phenogroups and those who did not, baseline demographic, clinical, laboratory and echocardiographic data were compared, with SMDs used to quantify the magnitude of between-group differences. Multivariable logistic regression, presented as OR with 95% CIs, was also performed with continuous variables standardised to identify baseline characteristics independently associated with phenogroup transition.

### External replication

We assessed phenogroup reproducibility by applying the same LCA approach to an independent external cohort from GSTT derived using identical inclusion criteria and NLP methods. Baseline demographic, clinical and echocardiographic characteristics of the resulting GSTT phenogroups were compared with those of the KCH derivation cohort. To quantitatively assess phenogroup reproducibility, the KCH-derived RF classifier was applied directly to the GSTT cohort without refitting and C-statistics were calculated using a one-versus-rest approach comparing RF-predicted phenogroup probabilities against independent GSTT LCA phenogroup labels.

### Statistical analysis

Baseline continuous variables were reported as mean±SD, or median with IQR; categorical data were summarised as counts and percentages. Continuous variables were compared with a t‐test or Kruskal-Wallis test, categorical variables were compared with a χ^2^ test. To account for death as a competing event for phenogroup transition, we defined outcomes as follows: transitions were defined as any change from baseline phenogroup to a different phenogroup where the patient remained alive in the new phenogroup at last observation; death without transition was defined as death occurring while still classified in the baseline phenogroup, treated as a competing event that precludes transition; censoring occurred for patients remaining in their baseline phenogroup who were alive at last follow-up. Event time was measured from baseline classification to the first occurrence of either transition or death, with censoring at last follow-up for patients remaining in their baseline phenogroup without experiencing either event. Competing risks regression using Fine-Gray subdistribution hazard (SHR) models estimated associations between baseline phenogroup and the probability of transition versus death without transition, with cumulative incidence functions computed for each competing event. Survival analysis employed Kaplan-Meier estimates with log-rank tests for univariate comparisons. Two Cox proportional hazards modelling approaches were used to assess the prognostic impact of phenogroup membership: a baseline model and a time-varying model treating current phenogroup as a time-dependent covariate. Both models were adjusted for age and sex. Model discrimination was quantified using Harrell’s C-index with 95% CIs. Model comparison was performed using Akaike Information Criterion (AIC) and likelihood ratio tests. P values <0.05 were designated statistically significant. To account for multiple comparisons across phenogroups in the Cox regression analyses, we applied the Benjamini-Hochberg procedure, controlling the false discovery rate at 5%. All analyses were performed using R V.4.4.0.

## Results

We analysed 2223 patients from our KCH discovery cohort, with a median age of 75 years (IQR 63–83) and 60% female. Comorbidities included hypertension (67%), CKD (33%), diabetes (26%), CAD (25%) and AF (23%). Median BMI was 29 kg/m² (IQR 25–34) and LVEF was 60.1% (IQR 58.1–62.5). Median follow-up was 4 years (IQR 2.2–6.1). LCA identified four distinct phenogroups, consisting of 703 (32%) elderly-atrial dysfunction, 503 (23%) cardiovascular-kidney-metabolic, 530 (24%) obesity-predominant and 487 (22%) young-low comorbidity patients at baseline (figure 1, table 1).

**Figure 1:**
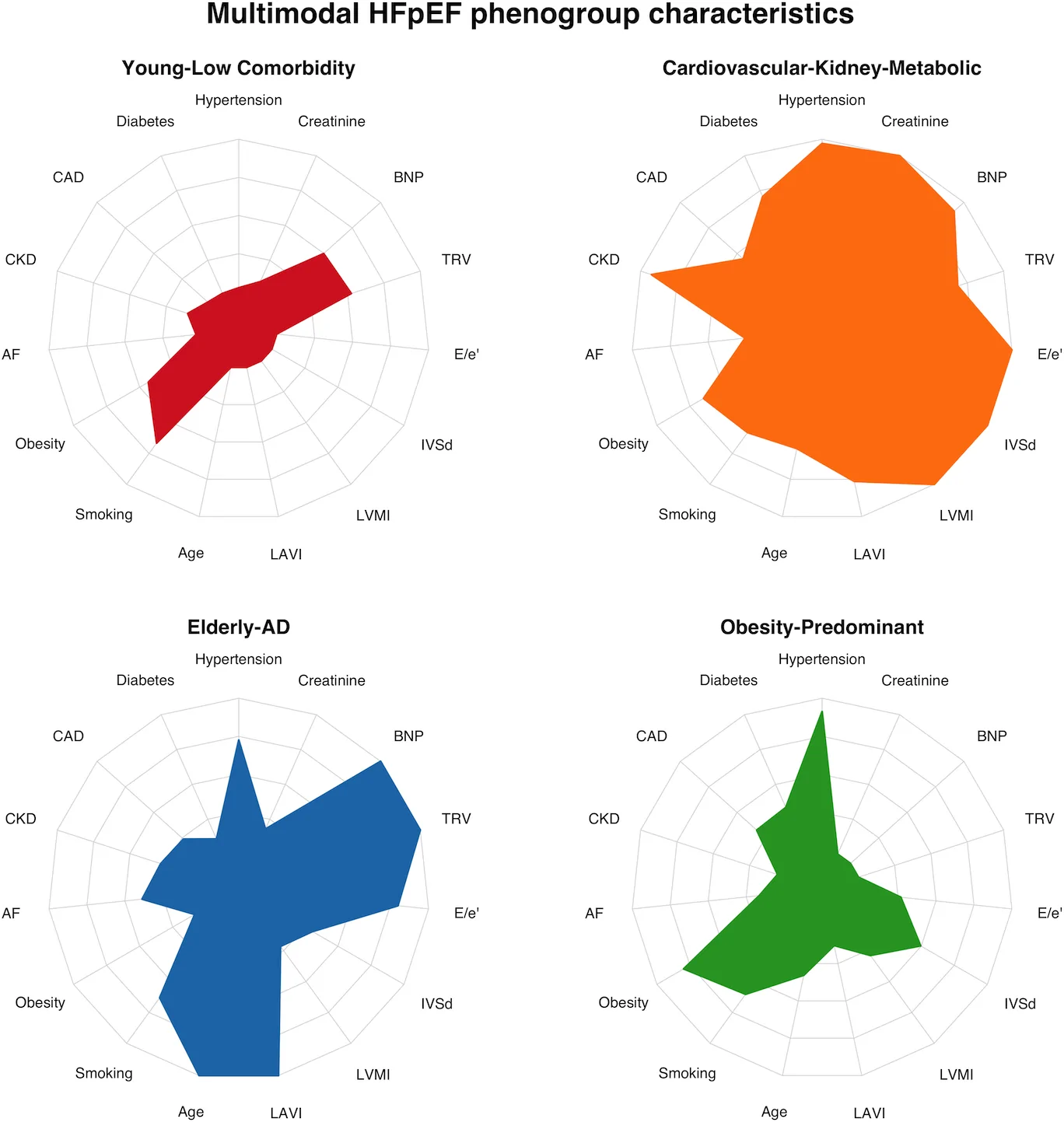
Multimodal HFpEF phenogroup characteristics. Radar plots depicting the relative differences in demographics, comorbidities, echocardiography variables and BNP across the four phenogroups: young-low comorbidity (red), obesity-predominant (green), elderly atrial dysfunction (AD) (blue) and cardiovascular-kidney-metabolic (orange). Categorical variables are shown as percentages, and continuous variables are displayed as normalised means of Z-standardised values. AD, atrial dysfunction; BNP, B-type natriuretic peptide; HFpEF, heart failure with preserved ejection fraction; IVSd, interventricular septum thickness at end-diastole; LAVI, left atrial volume index; LVMI, left ventricular mass index; TRV, tricuspid regurgitation maximal velocity.

**Table 1:** Baseline demographics, clinical and laboratory characteristics of HFpEF phenogroups

	Elderly atrial dysfunction, 32% (n=703)	Cardiovascular-kidney-metabolic, 23% (n=503)	Obesity predominant**,** 24% (n=530)	Young low comorbidity**,** 22% (n=487)	P value
Age	84 (81–88)	74 (65–81)	70 (61–76)	60 (48–71)	**<0.001**
Female	482 (69%)	253 (50%)	339 (64%)	257 (53%)	**<0.001**
Obesity	58 (8%)	321 (62%)	413 (78%)	209 (43%)	**<0.001**
BMI (kg/m^2^)	25 (22–28)	31 (28–36)	33 (30–38)	29 (25–34)	**<0.001**
Ethnicity					
White	484 (70%)	248 (48%)	257 (49%)	272 (55%)	**<0.001**
Black	101 (15%)	168 (33%)	181 (34%)	128 (26%)	**<0.001**
Asian	30 (4.4%)	46 (8.9%)	36 (6.9%)	22 (4.4%)	**0.003**
Hypertension	487 (69%)	480 (95%)	498 (94%)	14 (2.9%)	**<0.001**
Atrial fibrillation	264 (38%)	138 (27%)	95 (18%)	17 (3%)	**<0.001**
Cerebrovascular accident	174 (25%)	181 (35%)	102 (19%)	36 (7.3%)	**<0.001**
Chronic kidney disease	190 (27%)	498 (99%)	27 (5%)	48 (10%)	**<0.001**
Diabetes mellitus	70 (10%)	342 (68%)	176 (33%)	11 (2%)	**<0.001**
Pulmonary hypertension	189 (27%)	168 (33%)	144 (27%)	131 (27%)	**0.048**
Coronary artery disease	156 (22%)	218 (43%)	172 (32%)	18 (3.7%)	**<0.001**
Smoking history	453 (66%)	299 (58%)	297 (57%)	333 (67%)	**<0.001**
Anaemia	354 (50%)	305 (61%)	190 (36%)	252 (52%)	**<0.001**
Haemoglobin, g/L	121 (108–133)	117 (101–132)	130 (115–140)	122 (101–136)	**<0.001**
Creatinine, μmol/L	87 (70–114)	125 (90–203)	79 (64–96)	75 (59–97)	**<0.001**
Hypotensive medication	428 (61%)	448 (89%)	391 (74%)	100 (21%)	**<0.001**
Loop diuretics	316 (52%)	339 (75%)	219 (56%)	84 (29%)	**<0.001**
Beta blockers	267 (38%)	303 (60%)	206 (39%)	66 (14%)	**<0.001**
Calcium channel blockers	214 (30%)	336 (67%)	252 (48%)	25 (5%)	**<0.001**
ACE inhibitors	305 (43%)	377 (75%)	307 (58%)	46 (9%)	**<0.001**
NT-proBNP, pg/mL	3562 (2128–5349)	3824 (1506–7298)	987 (404–1853)	1775 (971–3134)	**<0.001**
H_2_FPEF score	6 (3–6)	5 (3–6)	4 (3–6)	3 (2–4)	**<0.001**
HFpEF-ABA score	81 (67–94)	81 (64–92)	75 (59–89)	51 (29–72)	**<0.001**

Median (IQR) or n (%).

### Phenogroup characteristics

Elderly-atrial dysfunction (median age 84 years) was predominantly female (69%) and characterised by marked atrial remodelling (median LA volume 42 mL/m²) with a high AF prevalence (38%) (tables 1 and 2). Cardiovascular-kidney-metabolic (median age 74 years) exhibited near universal renal dysfunction (99% CKD), predominant diabetes (68%), greatest LV hypertrophy (median LV mass 106 g/m², IQR 93–121) and highest NT-proBNP (3824 pg/mL). Obesity-predominant (median age 70 years) had the highest BMI (median 33 kg/m²) and number with hypertension (95%) but only moderate structural remodelling and the lowest NT-proBNP (987 pg/mL, IQR 404–1853). Young-low comorbidity (median age 60 years) exhibited few comorbidities (hypertension 3%, diabetes 2%) but elevated NT-proBNP (1775 pg/mL, IQR 971–3134) and fewer echocardiographic abnormalities. The LCA variables showing the largest between-phenogroup differences were hypertension (SMD 2.07), CKD (SMD 1.82), age (SMD 1.06) and diabetes (SMD 1.05) (online supplemental table S5). NT-proBNP (SMD 0.57), BMI (SMD 0.55), CAD (SMD 0.58) and AF (SMD 0.51) showed moderate discriminatory contribution.

**Table 2:** Echocardiographic parameters across HFpEF phenogroups

Characteristic	Elderly atrial dysfunction, n=703	Cardiovascular-kidney-metabolic, n=503	Obesity predominant**,** n=530	Young low comorbidity**,** n=487	P value
LVEF	59.6 (58.0–62.0)	60.9 (58.9–63.0)	60.4 (58.1–62.7)	59.7 (57.9–62.3)	**<0.001**
LV mass index (g/m^2^)					**<0.001**
Male	99 (89–116)	110 (95–127)	98 (84–110)	91 (78–106)	
Female	93 (82–104)	105 (92–120)	90 (80–101)	85 (73–98)	
LA volume index (mL/m^2^)					**<0.001**
Male	43 (37–51)	39 (33–47)	33 (28–39)	30 (27–37)	
Female	41 (33–48)	41 (34–46)	32 (27–38)	30 (27–37)	
Septal E/e’ ratio	15.4 (11.8–19.0)	15.5 (11.8–19.3)	12.2 (9.7–15.1)	10.7 (8.4–13.5)	**<0.001**
Average septal-lateral E/’e ratio	13.7 (10.9–17.0)	13.2 (10.0–17.0)	11.2 (8.8–13.9)	9.6 (7.5–12.1)	**<0.001**
Max TR velocity (cm/s)	2.81 (2.50–3.19)	2.72 (2.48–3.06)	2.57 (2.34–2.82)	2.67 (2.37–3.02)	**<0.001**
ePASP (mm Hg)	31 (25–41)	30 (25–38)	26 (22–32)	28 (22–37)	**<0.001**
IVSd (cm)	1.13 (1.00–1.29)	1.26 (1.09–1.43)	1.16 (1.02–1.31)	1.07 (0.93–1.24)	**<0.001**
LVPWd (cm)	1.10 (0.93–1.20)	1.20 (1.00–1.30)	1.10 (0.97–1.20)	1.00 (0.90–1.20)	**<0.001**
LVIDs (cm)	2.66 (2.42–2.93)	2.88 (2.60–3.18)	2.90 (2.62–3.25)	2.91 (2.63–3.22)	**<0.001**
LVEDD (cm)	4.20 (3.90–4.60)	4.50 (4.12–4.90)	4.53 (4.20–4.90)	4.50 (4.15–4.90)	**<0.001**

Median (IQR) or n (%).

ePASP, Estimated pulmonary artery systolic pressure; HFpEF, heart failure with preserved ejection fraction; IVSd, interventricular septum thickness at end-diastole; LA, left atrial; LV, left ventricle; LVEDD, Left ventricular end-diastolic diameter; LVEF, Left ventricular ejection fraction; LVIDs, Left ventricular internal diameter end diastole; LVPWd, Left ventricular posterior wall end diastole; TR, tricuspid regurgitation.

### External replication

In an independent external cohort of 3349 patients (median age 74 (IQR 61–82) years, 54% female), phenogroup reproducibility was demonstrated despite differences in baseline comorbidity prevalence (diabetes 36% vs 26%, AF 42% vs 23%, CKD 43% vs 33%). LCA identified four clusters with similar clinical characteristics and mortality (online supplemental table S6), (online supplemental figure S2). Against independently derived GSTT LCA phenogroup labels, the KCH RF model achieved C-statistics of 0.950 (95% CI 0.943 to 0.957) for elderly-atrial dysfunction, 0.906 (95% ci 0.895 to 0.917) for young-low comorbidity, 0.953 (95% ci 0.947 to 0.959) for cardiovascular-kidney-metabolic and 0.891 (95% ci 0.878 to 0.904) for obesity-predominant (online supplemental table S7), with overall agreement of 73.0% between RF-predicted and independently-derived GSTT LCA labels (online supplemental table S8).

### Phenogroup transitions

Over 8892 person-years of follow-up, 810 deaths occurred and 530 (23.8% of cohort) phenogroup transitions were observed. The RF model demonstrated high classification performance, with a mean prediction confidence of 0.92±0.11; 73% of assignments were made with >90% confidence. A stability analysis showed 92% phenotype consistency among patients with <10% change in clinical characteristics. The most important discriminative variables were hypertension, age and CKD, accounting for 54.1% of total variable importance (online supplemental figure S3).

Phenogroup trajectories diverged over time, distinguishing progressive phenotypes from more stable, end-stage groups (figure 2). The young-low comorbidity group demonstrated the greatest movement, with 47.7% (n=231) transitioning to new phenogroups over 5 years (online supplemental table S9), while competing mortality without transition was rare (0.4%, n=2) (online supplemental table S10). Transitions occurred predominantly within the first two years and were directed toward cardiovascular-kidney-metabolic (11.8%), obesity-predominant (11.2%) and elderly-atrial dysfunction phenogroups (13.8%). Although overall five-year mortality was 27.5% (n=133), nearly all deaths (98.5%) occurred after phenogroup transition. Compared with those who remained in the young-low comorbidity phenotype, the 231 who subsequently transitioned to another phenogroup had greater evidence of diastolic dysfunction and cardiac remodelling at baseline, with higher average E/e′ (SMD 0.67), interventricular septal thickness at end-diastole (IVSd) (SMD 0.64), LV mass index (SMD 0.58) and LA volume index (SMD 0.56) (online supplemental table S11). In contrast, baseline differences in conventional comorbidities, including AF, CKD, diabetes, hypertension and CAD, were small (SMDs 0.00–0.18). On multivariable analysis, older age, higher BMI, E/e′ ratio, IVSd, creatinine and AF were independently associated with subsequent transition from the young-low comorbidity phenotype (online supplemental figure S4).

**Figure 2:**
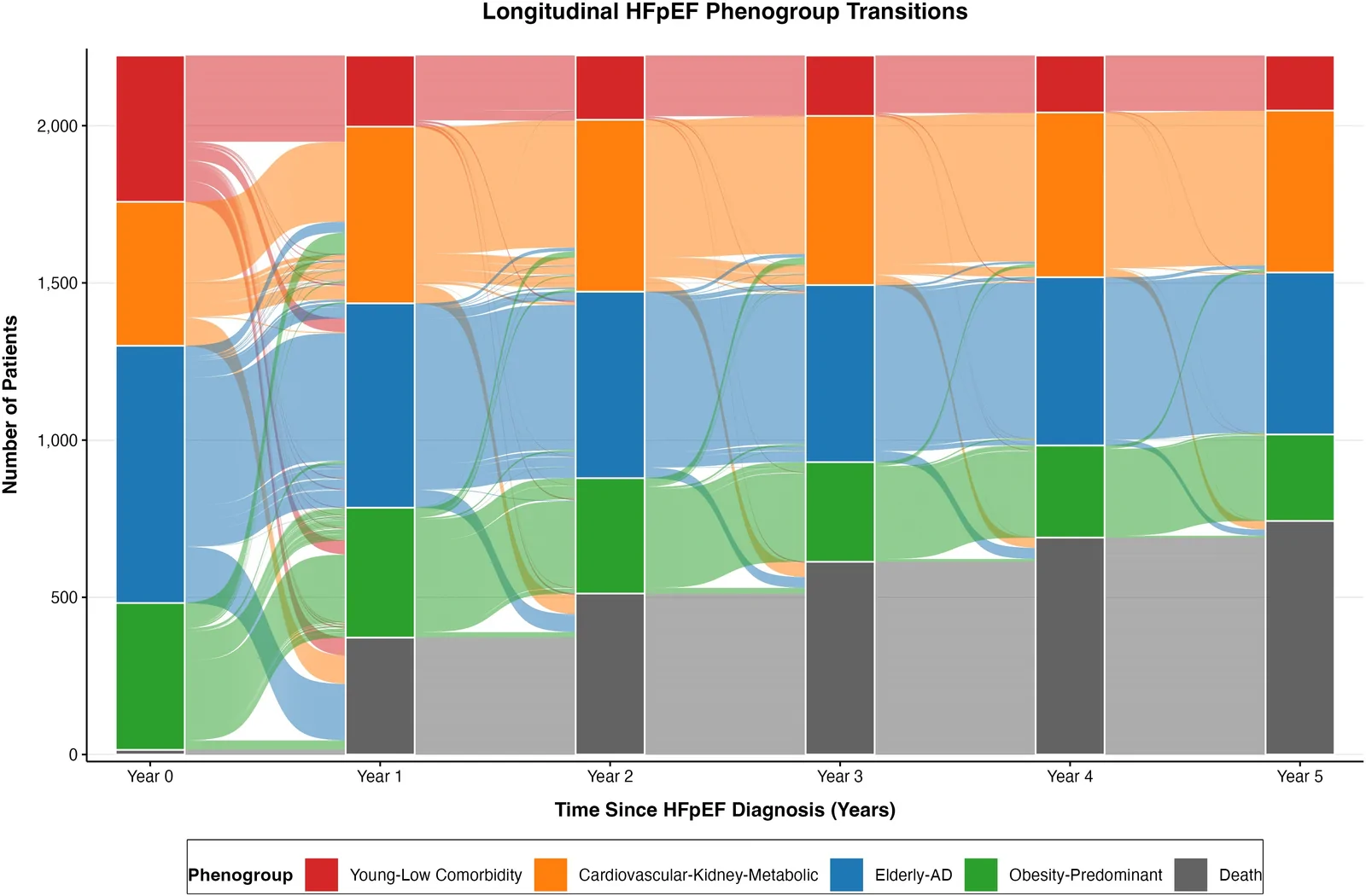
Longitudinal HFpEF phenogroup trajectories. Alluvial diagram depicting phenogroup transitions from baseline to 5 years. Each coloured stream represents a phenogroup colour; young-low comorbidity (red), obesity predominant (green), elderly atrial dysfunction (blue) and cardiovascular-kidney-metabolic (orange). Stream width is proportional to the number of patients. Diverging streams indicate transitions between phenogroups; converging streams show patients from multiple phenogroups transitioning to a common group. Death (grey) is shown as a competing outcome at each timepoint. AD, atrial dysfunction; HFpEF, heart failure with preserved ejection fraction.

The obesity-predominant phenogroup demonstrated intermediate mobility, with 39.3% (n=148) transitioning over follow-up, primarily toward the cardiovascular-kidney-metabolic phenotype (25.7%). In contrast, cardiovascular-kidney-metabolic and elderly-atrial dysfunction phenogroups displayed characteristics of advanced disease, with high stability and mortality. Cardiovascular-kidney-metabolic exhibited minimal transition to alternative phenogroups (3.2%), with 94.0% remaining stable throughout follow-up and a 5-year mortality of 45.6%, most occurring without phenogroup transition (93.9%). Similarly, elderly-atrial dysfunction showed limited phenotypic mobility (12.1%), with 85.1% remaining stable over time.

Fine-Gray SHR models confirmed substantially lower transition probabilities in advanced phenogroups compared with young-low comorbidity. Cardiovascular-kidney-metabolic demonstrated a 96% lower transition probability (SHR 0.04, 95% CI 0.02 to 0.07, p<0.001), Elderly-atrial dysfunction an 88% lower probability (SHR 0.12, 95% CI 0.09 to 0.15, p<0.001), and obesity-predominant a 36% lower probability (SHR 0.64, 95% CI 0.54 to 0.77, p<0.001).

### Phenogroup mortality outcomes

Overall 5-year survival was 64.0% (95% CI 62 to 66%), with phenogroup-specific mortality ranging from 26% in young-low comorbidity to 55% in cardiovascular-kidney-metabolic (figure 3A, (online supplemental table S12). In baseline-adjusted analyses, cardiovascular-kidney-metabolic was associated with a 53% higher mortality risk compared with young-low comorbidity (HR 1.53, 95% CI 1.22 to 1.92, p<0.001), whereas elderly atrial dysfunction (HR 1.21, 95% CI 0.95 to 1.53, p=0.12) and obesity-predominant phenogroups (HR 0.79, 95% CI 0.62 to 1.02, p=0.07) were not independently associated with mortality (figure 3B). In time-varying analyses incorporating longitudinal phenogroup transitions, current classification as cardiovascular-kidney-metabolic remained strongly associated with mortality (HR 1.72, 95% CI 1.31 to 2.27, p<0.001), while elderly-atrial dysfunction also demonstrated increased risk (HR 1.36, 95% CI 1.02 to 1.81, p=0.04). Obesity-predominant remained unassociated with mortality. Associations were independent of age and sex, and remained significant following false discovery rate correction. Compared with a baseline-only model (C-index 0.648, 95% CI 0.629 to 0.667), the time-varying model demonstrated modestly improved discrimination for mortality (C-index 0.659, 95% CI 0.638 to 0.679), with improved overall model fit (likelihood ratio test, ΔAIC=−203.4, p<0.001).

**Figure 3:**
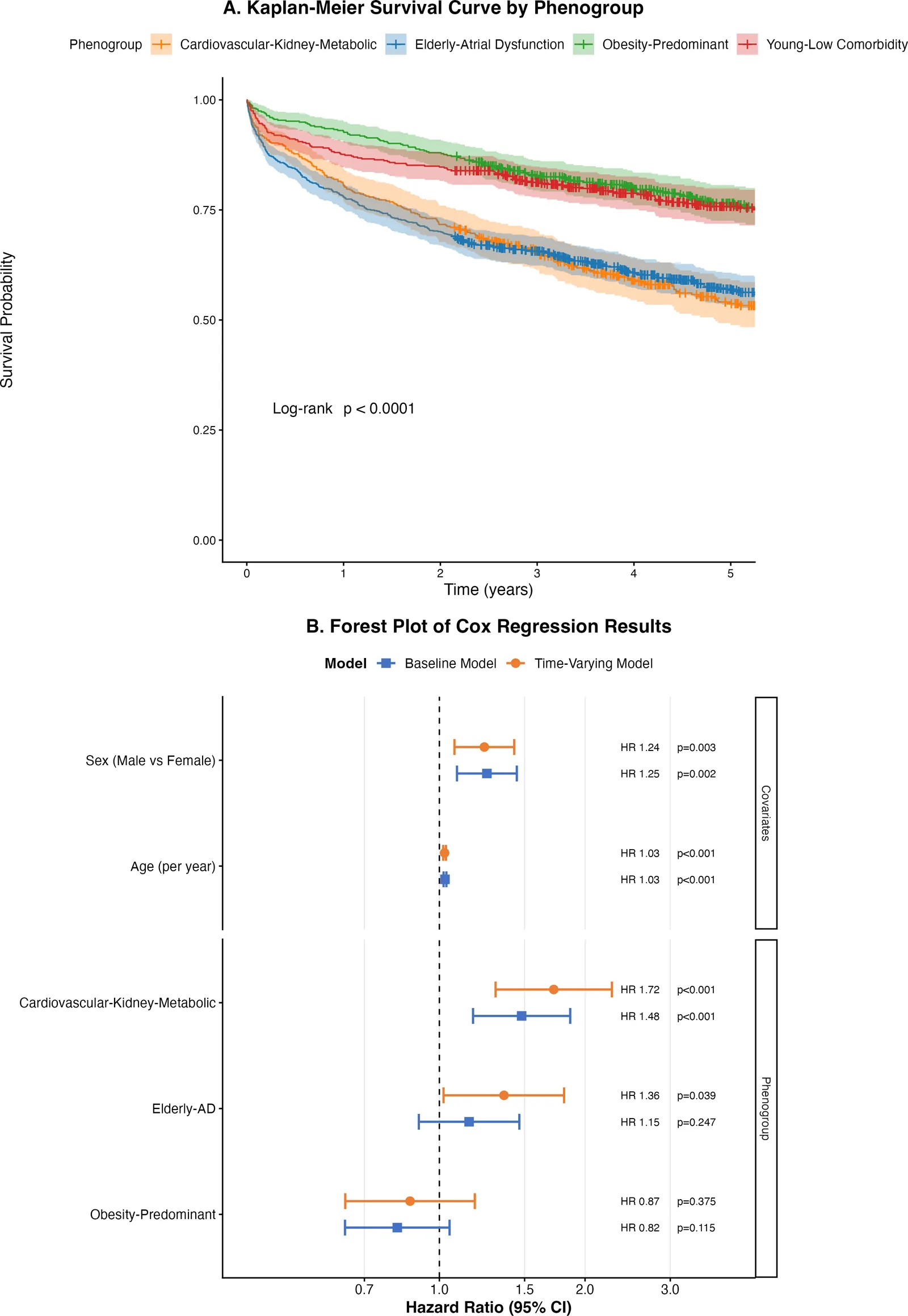
Kaplan-Meier survival curves and forest plot of adjusted HRs for HFpEF phenogroups. (**A**) Kaplan-Meier survival curves illustrating five-year survival estimates stratified by HFpEF phenogroup with log-rank test assessing survival differences. Phenogroups are colour-coded as: young-low comorbidity (red), obesity-predominant (green), elderly-atrial dysfunction (blue), and cardiovascular-kidney-metabolic (orange). (**B**) Forest plot displaying baseline and time-varying Cox proportional hazards models for mortality risk. HRs with 95% CIs are shown for each phenogroup and covariate. Baseline model (blue squares) represents phenogroup assignment at study entry; time-varying model (orange circles) represents current phenogroup status updated throughout follow-up. Both models are adjusted for age and sex. Young-low comorbidity serves as the reference group.

## Discussion

This study makes three key advances in understanding the heterogeneity and longitudinal evolution of real-world HFpEF. First, we identify a young, early-stage phenogroup with minimal comorbidities but mortality risk disproportionate to its comorbidity burden, challenging the conventional view of HFpEF as predominantly a disease of elderly, multimorbid patients. Second, we demonstrate that HFpEF phenogroups are dynamic rather than fixed, with nearly half of young-low comorbidity patients transitioning to higher-risk phenotypes over five years. Third, tracking phenogroup status longitudinally predicted mortality more accurately than baseline classification alone. Prior phenotyping studies have been largely cross-sectional,^7–9 12–14^ leaving phenotype stability and progression unexplored.^15^ Repeated machine learning classification applied to time-updated EHR data shows that phenogroup membership follows clinically meaningful trajectories, distinguishing progressive early-stage disease from stable, advanced phenotypes.

### Comparison with prior phenotyping studies

Our four phenogroups demonstrate strong concordance with prior HFpEF clustering studies despite important methodological differences. Uijl *et al*’s landmark analysis of the Swedish Heart Failure Registry identified five clusters including ‘older-atrial fibrillation’ (24.7%), ‘young with a low comorbidity burden’ (10.0%) and ‘cardio-renal’ (19.8%) phenotypes,^7^ while Segar *et al* delineated broadly similar phenotypes within the TOPCAT trial.^8^ Notably, the low-comorbidity phenogroup represented only 10% of patients in Uijl *et al*’s registry-based analysis compared with 22% in our NLP-derived cohort, suggesting that conventional trial and registry recruitment may systematically under-represent earlier-stage HFpEF. Unlike these predominantly cross-sectional analyses, our EHR cohort, identified using an NLP pipeline rather than clinician-coded diagnoses, enabled longitudinal tracking of phenogroup transitions in a real-world population, directly addressing the limitation highlighted by Peters *et al.*^15^

### Early-stage progressive HFpEF

The young-low comorbidity group appears to represent an early-stage HFpEF phenotype, characterised by relatively mild structural and functional abnormalities despite elevated NT-proBNP. Although comorbidity burden was low at baseline, nearly half transitioned to higher-risk phenogroups within five years, with most deaths occurring after phenogroup transition, suggesting that phenotypic progression precedes mortality. Notably, patients who subsequently transitioned had more abnormal echocardiographic features at baseline, including higher E/e′, IVSd, LV mass and LA volume. In multivariable analysis, both E/e′ ratio and IVSd remained independently associated with subsequent transition. These findings suggest that phenotypic progression may be preceded by identifiable cardiac abnormalities, potentially providing an opportunity to identify patients at greater risk of progression. This phenotype is analogous to the low-comorbidity subgroups described by Kao *et al* and Segar *et al*,^8 14^ comprising younger patients with fewer traditional risk factors despite objective evidence of HFpEF; the median age of 60 years falls within the accepted HFpEF age distribution.^22^

The obesity-predominant phenogroup demonstrated intermediate mobility, with transitions occurring predominantly towards the cardiovascular-kidney-metabolic phenotype. This progression likely reflects the well-described natural history of obesity-related HFpEF,^25^ in which accumulating metabolic burden drives progressive cardiac remodelling and, ultimately, cardiorenal syndrome. Notably, those patients remaining stable in this phenotype had mortality comparable to the young-low comorbidity group, suggesting that accumulating cardiometabolic burden is the principal determinant of adverse outcomes within this subgroup.

### Stable end-stage HFpEF

The elderly atrial dysfunction and cardiovascular-kidney-metabolic phenogroups appear to represent two dominant pathophysiological pathways within advanced HFpEF. These phenotypes demonstrated the greatest stability and highest mortality throughout follow-up, with limited movement to alternative phenogroups, consistent with an established disease state. This is in contrast to the young-low comorbidity group, in whom nearly all deaths followed transition. This distinction is directly supported by the time-varying Cox models: current classification as cardiovascular-kidney-metabolic or elderly atrial dysfunction predicted mortality more accurately than baseline phenogroup assignment alone, with current cardiovascular-kidney-metabolic and elderly atrial dysfunction status conferring 72% and 36% excess mortality risk respectively, relative to young-low comorbidity. Although longitudinal phenogroup status provided modestly greater mortality discrimination than baseline classification, the absolute C-index remained modest (0.659) within the range reported for other scores used in HFpEF.^26^ This indicates that while phenogroup captures clinically relevant prognostic information it should not be used alone for mortality prediction.

The elderly atrial dysfunction phenotype’s advanced age, severe atrial remodelling and high AF burden support atrial myopathy as a distinct mechanistic pathway in advanced HFpEF.^4 27^ In parallel, the cardiovascular-kidney-metabolic phenotype, which was characterised by near-universal renal dysfunction, predominant diabetes and marked LV hypertrophy, is consistent with cumulative cardiorenal and metabolic injury driving progressive myocardial dysfunction. Together, these phenotypes define the end of the HFpEF disease spectrum.

### Clinical implications and future directions

These findings have several clinical and research implications. First, the young-low comorbidity phenogroup carries mortality comparable to the more established obesity-predominant phenotype despite fewer traditional risk factors, highlighting the potential importance of earlier recognition and management. Recent trials demonstrate that sodium-glucose cotransporter 2 inhibitors and glucagon-like peptide 1 receptor agonists improve HFpEF outcomes,^28 29^ yet trial populations have predominantly comprised older, multimorbid patients. Whether early initiation of these therapies in younger, low-comorbidity patients can alter subsequent phenotypic progression and structural remodelling remains an important question for future studies.

Taken together, our EHR-based approach supports the development of automated, AI-driven diagnostic tools for routine clinical use,^18^ enabling proactive identification of HFpEF in high-risk populations, particularly those with obesity or AF,^3 4^ before advanced structural disease is established. This shifts the management from late-stage symptom relief towards earlier, phenotype-targeted intervention.

### Strengths and limitations

Strengths include the use of NLP to identify HFpEF directly from unstructured EHR text, which broadens diagnostic capture beyond clinician-coded registries. The study also incorporates longitudinal tracking of individual phenogroup transitions using repeated machine learning classification, which to our knowledge, is the first analysis of its kind in HFpEF. Another key strength is the two-step approach to external reproducibility. By applying the KCH-derived RF classifier directly to the GSTT cohort without refitting, we provided a quantitative test of whether a model trained entirely on one population generalises to another. The C-statistics of 0.891–0.953 provide evidence that the phenogroup structure was reproducible in an independent real-world HFpEF population, comparable to the range reported for cross-dataset cluster prediction in other EHR-derived phenotyping studies (C-statistics 0.79–0.94).^30^

We acknowledge several limitations. First, this was a retrospective study, and although findings were externally replicated, the external cohort characteristics were substantially different in several defining features, therefore validation in other geographical populations is needed. Second, reliance on EHR-based NLP extraction depends on the completeness and accuracy of routine clinical documentation and assumes that patients maintained contact with our institution throughout the follow-up period. Patients who received care elsewhere may have experienced clinical changes not captured in the EHR systems, potentially leading to under-recording or misclassification and therefore underestimating our observed transitions. Third, cause-specific mortality data were unavailable, limiting the ability to distinguish cardiovascular from non-cardiovascular deaths. Fourth, treatment data were limited to medication classes without information on dose, adherence or changes during follow-up. Whether phenotype transitions were modified by therapeutic interventions cannot be determined from our observational data. Finally, clustering was based primarily on comorbidity and core clinical data; inclusion of additional biomarkers, imaging or genomic information in future work may refine phenogroup definitions.

### Conclusion

Four reproducible HFpEF phenogroups were identified from real-world EHR data using NLP-driven phenotyping, with their structure replicated in an independent cohort. Longitudinal analysis revealed that phenogroup membership is dynamic, with young-low comorbidity patients frequently transitioning towards higher-risk phenotypes over 5 years, while advanced phenogroups remained stable with the highest mortality. Earlier HFpEF detection using AI-driven EHR tools could facilitate phenotype-targeted treatment of patients at potentially modifiable stages.

## Supplementary material

10.1136/openhrt-2026-004351 online supplemental file 1

## Data Availability

Data are available upon reasonable request.
