# Supplementary material for: Longitudinal phenotyping of heart failure with preserved ejection fraction identifies early- and end-stage disease states
Source: Open Heart. 2026 Sep 25;13(2):e004351. doi: 10.1136/openhrt-2026-004351 (PMC13630162; doi:10.1136/openhrt-2026-004351)
Supplement: Supplementary file 1 [file openhrt-13-2-s001.docx]

**Supplemental material**

| **Contents** | **Page number(s)** |
| --- | --- |
| Supplemental Methods S1 | 2 |
| Supplemental Table S1 | 5 |
| Supplemental Table S2 | 6 |
| Supplemental Table S3 | 7-8 |
| Supplemental Table S4 | 9 |
| Supplemental Table S5 | 10 |
| Supplemental Table S6 | 11-12 |
| Supplemental Table S7 | 13 |
| Supplemental Table S8 | 13 |
| Supplemental Table S9 | 14 |
| Supplemental Table S10 | 15 |
| Supplemental Table S11 | 16 |
| Supplemental Table S12 | 17 |
| Supplemental Figure S1 | 18 |
| Supplemental Figure S2 | 19 |
| Supplemental Figure S3 | 20 |
| Supplemental Figure S4 | 21 |

**Supplemental Methods S1:**

**HFpEF Cohort Identification using Natural Language Processing (NLP)**

The CogStack information retrieval system deployed at the King’s College Hospital NHS Foundation Trust collects structured and unstructured routinely collected data within the hospital.^1^ Structured data includes demographic (date of birth, sex, ethnicity, laboratory observations, etc) while unstructured data include free text documents of different types (clinical notes, discharge notifications, referral letters, etc). CogStack enables a single point of information retrieval related to patients’ electronic health records.

To facilitate effective and efficient search of clinical concepts in the unstructured data (free text documents), Medical Concept Annotation Toolkit (MedCAT) is employed to annotate the text documents for all SNOMED-CT (Systematized Nomenclature of Medicine - Clinical Terms) concepts using machine learning approach.^2^ SNOMED-CT is a widely used structured clinical vocabulary for electronic health records. Every SNOMED-CT concept represents a unique clinical meaning, which is referenced using a unique, numeric code and identifier, and has unique relationships with other concepts (e.g. subtypes of a disease). MedCAT is trained in both supervised and unsupervised fashion to achieve good accuracy for the detection of clinical concepts (F1 score > 0.9).^3,4^ MedCAT uses a novel concept disambiguation algorithm that learns clinical concept similarity via Word vector contexts and employs a Bidirectional Long-Short-Term-Memory (Bi-LSTM) model, a kind of deep learning models, for contextualisation (e.g., experiencer, negation, and temporal information). Correction of spelling mistakes is also performed during the annotation process.

MedCAT produced unsupervised annotations for all SNOMED‐CT concepts under parent terms Clinical Finding, Disorder, Organism, and Event with disambiguation, pre‐trained on Medical Information Mart for Intensive Care III (MIMIC-III), a large, de-identified and publicly-available dataset. Further supervised training improved detection of annotations and meta‐annotations such as experiencer (is the concept annotated experienced by the patient or other), negation (is the concept annotated negated or not) and temporality (is the concept annotated in the past or present) with MedCATTrainer.^5^ Meta‐annotations for hypothetical and experiencer were merged into irrelevant meaning that any concept annotated as either hypothetical or where the experiencer was not the patient was annotated as irrelevant. Performance of the MedCAT NLP pipeline for disorders mentioned in the text was evaluated on more than 5600 annotations for >265 documents by a domain expert and F1, precision and recall recorded.

**HFpEF Cohort Identification**

Two cohorts were identified from NLP and combined. First, a clinician-documented HFpEF cohort with the SNOMED-CT Term “Heart failure with normal ejection fraction” (ID: 446221000) and any of its child terms were identified and then then the ESC HFpEF cohort were identified, requiring all three of.

1. Heart failure (disorder) SNOMED-CT Term (ID: 84114007) captured more than twice.
2. LVEF ≥50% within a year of the HF mention
3. Imaging/biochemical evidence of diastolic dysfunction meeting the ESC diagnostic criteria. This includes one of: raised NT-proBNP ≥125 pg/mL (≥375 pg/mL with atrial fibrillation [AF]), LV mass index ≥95 g/m (female) 115 g/m^2^ (male), relative wall thickness >0.42, left atrial volume index >34 mL/m^2^ (>40 mL/m^2^ with AF), Doppler echocardiographic E/e’ (ratio of early diastolic mitral inflow velocity to mitral annulus relaxation velocity) ratio >9, pulmonary artery systolic pressure (PASP) >35 mm Hg, or tricuspid regurgitation velocity >2.8 m/s.

Patients were excluded if at any point they had an echocardiogram with LVEF <50%. Patients were excluded if there was an alternative diagnosis documented including hypertrophic cardiomyopathy, restrictive cardiomyopathy, constrictive pericarditis, cardiac amyloidosis, or severe valvular disease (see table below). Where a criterion is indexed to body surface area (i.e., left ventricular mass index, left ventricular volume index) but where no indexed value could be found in the EHR, we collected data for the raw value and applied these values (i.e. left ventricular mass in grams, left atrial volume in ml).

SNOMED-CT terms used in the study for identifying the HF + LVEF ≥ 50% patients and the subgroups. All child terms are included by default.

| **Patient Group** | **SNOMED-CT Term** | **ID** |
| --- | --- | --- |
| HF patients | Heart failure (disorder) | 84114007 |
| Clinician-documented HFpEF patients | Heart failure with normal ejection fraction (disorder) | 446221000 |
| Alternative Diagnosis patients | Aortic valve stenosis (disorder)^*^ | 60573004 |
|  | Aortic valve regurgitation (disorder) ^*^ | 60234000 |
|  | Mitral valve stenosis (disorder) ^*^ | 79619009 |
|  | Mitral valve regurgitation (disorder) ^*^ | 48724000 |
|  | Pulmonic valve stenosis (disorder) ^*^ | 56786000 |
|  | Pulmonic valve regurgitation (disorder) ^*^ | 91434003 |
|  | Hypertrophic cardiomyopathy (disorder) | 233873004 |
|  | Senile cardiac amyloidosis (disorder) | 16573007 |
|  | Restrictive cardiomyopathy (disorder) | 415295002 |
|  | Constrictive pericarditis (disorder) | 415295002 |

* - the **severe** valvular heart disease patients are identified with the ‘severe’ keyword before the mentions of the SNOMED-CT terms.

**Supplemental Table S1.** Missing variable counts and percentages

|  | Missing Count | Missing Percent |
| --- | --- | --- |
| Age | 0 | 0 |
| Sex | 0 | 0 |
| Atrial Fibrillation | 0 | 0 |
| Diabetes Mellitus | 0 | 0 |
| CVA | 0 | 0 |
| Hypertension | 0 | 0 |
| Chronic kidney disease | 0 | 0 |
| Ischaemic Heart Disease | 0 | 0 |
| Myocardial infarction | 0 | 0 |
| Smoking status | 0 | 0 |
| ACEi/ARBs | 0 | 0 |
| Beta-blockers | 0 | 0 |
| Calcium channel blockers | 0 | 0 |
| Loop diuretics | 0 | 0 |
| Body Mass Index | 450 | 20.3 |
| IVSd | 96 | 4.3 |
| LVIDs | 689 | 31.0 |
| LVEDD | 359 | 16.1 |
| LVPWd | 366 | 16.5 |
| LV mass | 99 | 4.5 |
| LA volume | 673 | 30.3 |
| MV E max vel | 320 | 14.4 |
| MV A max vel | 634 | 28.5 |
| E/e' Lateral | 551 | 24.8 |
| E/e' Septal | 524 | 23.6 |
| TR max vel | 367 | 16.5 |
| Haemoglobin | 6 | 0.3 |
| Creatinine | 4 | 0.2 |
| HbA1c | 1254 | 56.4 |
| Natriuretic Peptide z-score | 688 | 30.9 |

**Supplemental Table S2.** BIC values per number of classes in LCA model

| **Classes** | **BIC** |
| --- | --- |
| 2 | 33655.96 |
| 3 | 33309.68 |
| 4 | 33230.46 |
| 5 | 33247.81 |
| 6 | 33271.72 |
| 7 | 33311.56 |
| 8 | 33373.37 |
| 9 | 33450.25 |
| 10 | 33524.63 |

Abbreviations: BIC, Bayesian information criterion

**Supplemental Table S3.** **Class Probabilities by Comorbidity Level.** The partial probability of the presence of an indicator variable in each latent class, which highlights the probability of that variable being present in that phenogroup. Partial probabilities were averaged over the 20 replications and then used to calculate each group membership. The conditional probability of the presence of an indicator variable in each latent class which highlights the probability of a variable being present in that phenotype. Abbreviations: BMI, Body Mass Index CAD, coronary artery disease; CKD, chronic kidney disease.

| **Variable** | **Elderly-Atrial Dysfunction,**  N = 703 | **Cardiovascular-Kidney-Metabolic**,  N = 503 | **Obesity-Predominant**,  N = 530 | **Young-Low Comorbidity**,  N = 487 |
| --- | --- | --- | --- | --- |
| Age < 60 years | 0.02271893 | 0.13228046 | 0.20851278 | 0.46894265 |
| Age 60 - 70 | 0.04968999 | 0.20745171 | 0.26057818 | 0.21685732 |
| Age 70 - 80 | 0.16191400 | 0.32446884 | 0.38242536 | 0.21647623 |
| Age > 80 years | 0.77114742 | 0.34071361 | 0.14848368 | 0.09015402 |
| Sex Female | 0.67700233 | 0.50725956 | 0.65085457 | 0.53378617 |
| Sex Male | 0.32299767 | 0.49274044 | 0.34914543 | 0.46621383 |
| BMI < 18.5 | 0.08847939 | 0.01107723 | 0.04360472 | 0.02002469 |
| BMI 18.5-24.9 | 0.39536476 | 0.10798618 | 0.07332654 | 0.18908790 |
| BMI 25-29.9 | 0.41455949 | 0.29194992 | 0.18061452 | 0.33281751 |
| BMI > 30 | 0.10159637 | 0.58898667 | 0.70245423 | 0.45806990 |
| Atrial Fibrillation No | 0.63296953 | 0.71307368 | 0.80197069 | 0.95557702 |
| Atrial Fibrillation Yes | 0.36703047 | 0.28692632 | 0.19802931 | 0.04442298 |
| CKD No | 0.72270168 | 0.16860183 | 0.87085605 | 0.89091803 |
| CKD Yes | 0.27729832 | 0.83139817 | 0.12914395 | 0.10908197 |
| Diabetes No | 0.89651793 | 0.36600523 | 0.68101738 | 0.96909833 |
| Diabetes Yes | 0.10348207 | 0.63399477 | 0.31898262 | 0.03090167 |
| Hypertension No | 0.31686791 | 0.05330707 | 0.10220330 | 0.88999739 |
| Hypertension Yes | 0.68313209 | 0.94669293 | 0.89779670 | 0.11000261 |
| CAD No | 0.77739196 | 0.56432987 | 0.69078495 | 0.94894748 |
| CAD Yes | 0.22260804 | 0.43567013 | 0.30921505 | 0.05105252 |
| Smoker No | 0.34327188 | 0.41353721 | 0.44076276 | 0.32208590 |
| Smoker Yes | 0.65672812 | 0.58646279 | 0.55923724 | 0.67791410 |
| NT-proBNP/BNP Q1 | 0.08132233 | 0.17118786 | 0.54030519 | 0.29354698 |
| NT-proBNP/BNP Q2 | 0.18945099 | 0.15959368 | 0.27545737 | 0.35471874 |
| NT-proBNP/BNP Q3 | 0.31928124 | 0.20584381 | 0.16389546 | 0.22718793 |
| NT-proBNP/BNP Q4 | 0.40994543 | 0.46337465 | 0.02034199 | 0.12454635 |

**Supplemental Table S4.** Maximum cluster probability

|  | **Overall,**  N = 2223 | **Elderly-Atrial Dysfunction,**  N = 703 | **Cardiovascular-Kidney-Metabolic**,  N = 503 | **Obesity-Predominant**,  N = 530 | **Young-Low Comorbidity**,  N = 487 |
| --- | --- | --- | --- | --- | --- |
| **Maximum cluster probability** | 85.3 (68.5-93.8) | 82.6 (67-93.8) | 85.2 (66.5-94.0) | 83.5 (67.5-91.3) | 90.2 (76.7-95.7) |
| **Maximum cluster probability >90%, %** | 37.6 | 35.5 | 35.4 | 29.8 | 51.2 |
| **Maximum cluster probability >70%, %** | 73.8 | 70.6 | 73.0 | 71.6 | 81.5 |
| **Maximum cluster probability >50%, %** | 94.3 | 92.6 | 95.4 | 93.1 | 97.0 |

**Supplemental Table S5.** Between-phenogroup differences in LCA variables, expressed as mean pairwise standardised mean difference (SMD) across all phenogroup pairs.

| **Variable** | **Used in LCA** | **Elderly-Atrial Dysfunction** | **Young-low Comorbidity** | **Cardio-Renal-Metabolic** | **Obesity-Predominant** | **Mean pairwise SMD** |
| --- | --- | --- | --- | --- | --- | --- |
| **Hypertension** | Yes | 580 (72.1) | 19 (3.9) | 438 (96.9) | 442 (92.9) | 2.07 |
| **CKD** | Yes | 223 (27.7) | 51 (10.4) | 435 (96.2) | 27 (5.7) | 1.81 |
| **Age at diagnosis** | Yes | 82.74 (7.77) | 58.83 (16.47) | 71.88 (12.00) | 66.68 (12.31) | 1.06 |
| **Diabetes** | Yes | 88 (10.9) | 7 (1.4) | 316 (69.9) | 171 (35.9) | 1.05 |
| **CAD** | Yes | 191 (23.8) | 15 (3.1) | 200 (44.2) | 158 (33.2) | 0.58 |
| **NT-proBNP** | Yes | 3440.94 (3700.48) | 1912.01 (2466.47) | 3962.52 (5050.88) | 943.84 (858.25) | 0.57 |
| **BMI** | Yes | 25.60 (12.41) | 29.53 (5.98) | 31.97 (5.80) | 33.81 (6.26) | 0.55 |
| HbA1c | No | 45.16 (8.87) | 45.48 (9.67) | 57.88 (19.35) | 51.18 (13.77) | 0.51 |
| **AF** | Yes | 305 (37.9) | 16 (3.3) | 116 (25.7) | 83 (17.4) | 0.51 |
| E/e' ratio (average) | No | 13.82 (5.29) | 10.38 (4.22) | 14.48 (5.44) | 11.68 (4.13) | 0.50 |
| E/e' ratio (septal) | No | 15.94 (5.92) | 11.75 (4.95) | 16.03 (6.32) | 12.87 (4.81) | 0.48 |
| LA volume index | No | 42.09 (13.89) | 32.99 (11.46) | 39.54 (10.58) | 34.46 (10.38) | 0.46 |
| Creatinine | No | 103.91 (86.90) | 97.96 (99.34) | 190.40 (174.89) | 89.95 (49.00) | 0.40 |
| IVSd | No | 1.16 (0.25) | 1.10 (0.26) | 1.27 (0.25) | 1.19 (0.23) | 0.36 |
| LV mass index | No | 96.04 (21.86) | 91.88 (25.77) | 108.98 (31.26) | 96.87 (23.66) | 0.32 |
| Systolic BP | No | 128.95 (14.11) | 124.79 (15.16) | 133.37 (16.86) | 131.29 (16.96) | 0.30 |
| **Sex** | Yes | 248 (30.8) | 227 (46.2) | 236 (52.2) | 181 (38.0) | 0.25 |
| TR max velocity | No | 286.60 (54.84) | 273.20 (59.44) | 276.53 (51.80) | 261.71 (49.09) | 0.24 |
| Haemoglobin | No | 120.78 (19.62) | 119.20 (23.39) | 116.65 (21.94) | 126.47 (20.47) | 0.24 |
| Estimated PASP | No | 34.07 (13.48) | 31.30 (14.16) | 31.72 (11.91) | 28.37 (11.19) | 0.23 |
| LVEF | No | 59.97 (4.16) | 60.17 (3.95) | 61.14 (4.76) | 60.64 (4.83) | 0.15 |

*Mean pairwise standardised mean differences (SMDs) for all latent class analysis (LCA) input variables and additional clinical characteristics, calculated across all six pairwise comparisons among the four phenogroups. Variables used to define the LCA model are indicated. SMD ≥0.5 was considered indicative of a moderate, and ≥0.8 a large, between-group difference.*

**Supplemental Table S6.** Baseline Demographics, Clinical, Laboratory and Echocardiography Characteristics of HFpEF Phenogroups for the external replication cohort (n = 3,349)

| **Characteristic** | **Elderly-AD**  **N = 735** | **Cardiovascular-Kidney-Metabolic**  **N = 1,217** | **Obesity**  **predominant**  **N = 676** | **Young-Low Comorbidity**  **N = 721** | **p-value** |
| --- | --- | --- | --- | --- | --- |
| Age (years) | 83 (77, 88) | 76 (67, 83) | 68 (61, 75) | 59 (47, 78) | <0.001 |
| Female | 467 (64%) | 636 (52%) | 240 (36%) | 475 (66%) | <0.001 |
| Obesity | 94 (13%) | 572 (47%) | 523 (77%) | 150 (21%) | <0.001 |
| BMI (Kg/m2) | 27.2 (25.7, 28.8) | 29.6 (26.9, 32.0) | 31.0 (30.0, 32.7) | 27.4 (24.8, 29.5) | <0.001 |
| Ethnicity |  |  |  |  | <0.001 |
| White | 508 (69%) | 665 (55%) | 392 (58%) | 385 (53%) |  |
| Asian | 10 (1.4%) | 72 (5.9%) | 21 (3.1%) | 25 (3.5%) |  |
| Black | 55 (7.5%) | 345 (24%) | 94 (12%) | 86 (17%) |  |
| NT-proBNP (pg/ml) | 3,687 (1,907, 6,040) | 2,039 (707, 4,120) | 928 (380, 1,651) | 2,116 (555, 4,825) | <0.001 |
| Atrial Fibrillation | 632 (86%) | 475 (39%) | 238 (35%) | 53 (7.4%) | <0.001 |
| Chronic Kidney Disease | 227 (31%) | 994 (82%) | 43 (6.4%) | 170 (24%) | <0.001 |
| Diabetes | 66 (9.0%) | 888 (73%) | 201 (30%) | 37 (5.1%) | <0.001 |
| Hypertension | 501 (68%) | 1,165 (96%) | 422 (62%) | 223 (31%) | <0.001 |
| Coronary Artery Disease | 148 (20%) | 603 (50%) | 203 (30%) | 41 (5.7%) | <0.001 |
| Pulmonary Hypertension | 89 (12%) | 123 (10%) | 45 (6.7%) | 75 (10%) | 0.03 |
| Average E/e’ ratio | 12.3 (9.2, 15.5) | 12.0 (9.2, 15.0) | 10.0 (8.0, 12.9) | 10.0 (8.0, 13.0) | <0.001 |
| IVSd (cm) | 1.19 (1.00, 1.30) | 1.20 (1.10, 1.38) | 1.20 (1.00, 1.30) | 1.10 (0.90, 1.30) | <0.001 |
| LA volume index (ml/m2) | 41 (34, 52) | 35 (29, 43) | 34 (29, 42) | 32 (26, 40) | <0.001 |
| LVEDD (cm) | 4.40 (3.90, 4.90) | 4.60 (4.10, 4.90) | 4.70 (4.30, 5.10) | 4.40 (3.90, 4.90) | <0.001 |
| LVEF % | 60 (55, 60) | 60 (55, 65) | 60 (55, 65) | 60 (55, 61) | <0.001 |
| LVPWd (cm) | 1.10 (0.90, 1.20) | 1.10 (1.00, 1.3) | 1.1 (0.90, 1.20) | 1.00 (0.90, 1.20) | <0.001 |
| LV mass (g) | 173 (137, 212) | 194 (157, 232) | 194 (157, 235) | 164 (123, 209) | <0.001 |
| LV mass index (g/m2) | 97 (82, 113) | 102 (87, 116) | 98 (85, 113) | 95 (74, 111) | <0.001 |
| ePASP (mmHg) | 31 (27, 39) | 29 (26, 33) | 28 (24, 31) | 29 (24, 39) | <0.001 |
| Max TR velocity (mm/s) | 263 (240, 300) | 260 (242, 279) | 248 (229, 269) | 253 (232, 300) | <0.001 |
| Loop diuretic use | 424 (58%) | 662 (54%) | 264 (39%) | 236 (33%) | <0.001 |
| H_2_FPEF score | 5 (5, 6) | 4 (2, 5) | 3 (2, 5) | 2 (1, 2) | <0.001 |
| H_2_FPEF probability of HFpEF | 0.90 (0.82, 0.94) | 0.75 (0.54, 0.90) | 0.63 (0.44, 0.81) | 0.44 (0.24, 0.68) | <0.001 |
| HFpEF-ABA probability | 0.95 (0.91, 0.97) | 0.81 (0.64, 0.94) | 0.73 (0.58, 0.90) | 0.52 (0.25, 0.72) | <0.001 |

*Data are presented as median (IQR) or n (%). Abbreviations: LVEF, Left ventricular ejection fraction; TR, tricuspid regurgitation; ePASP, Estimated pulmonary artery systolic pressure; IVSd, interventricular septal thickness end diastole; LVPWd, Left ventricular posterior wall end diastole; LVIDs, Left ventricular internal diameter end diastole; LVEDD, Left ventricular end-diastolic diameter.*

**Supplemental Table S7 & S8:** Application of Derivation Random Forest Classifier to GSTT External Replication Cohort

**Supplemental Table S7:** C-statistics for Random Forest classifier phenogroup prediction against independent GSTT LCA labels

| **Phenogroup** | **C-statistic** | **95% CI** |
| --- | --- | --- |
| Elderly-Atrial Dysfunction | 0.950 | 0.943–0.957 |
| Young-Low Comorbidity | 0.906 | 0.895–0.917 |
| Cardiovascular-Kidney-Metabolic | 0.953 | 0.947–0.959 |
| Obesity-Predominant | 0.891 | 0.878–0.904 |

**Supplemental Table S8:** Confusion Matrix. Random Forest -Predicted vs Independent GSTT LCA Labels

| **RF Predicted \ GSTT LCA** | **Elderly-AD** | **Young-Low Comorbidity** | **CKM** | **Obesity-predominant** |
| --- | --- | --- | --- | --- |
| Elderly-Atrial Dysfunction | **705** | 187 | 177 | 108 |
| Young-Low Comorbidity | 13 | **412** | 2 | 127 |
| Cardiovascular-Kidney-Metabolic | 10 | 51 | **899** | 12 |
| Obesity-Predominant | 7 | 71 | 139 | **429** |
| **Per-phenogroup agreement** | **95.9%** | **57.1%** | **73.9%** | **63.5%** |

*Cross-tabulation of phenogroup assignments from the KCH-derived random forest classifier against phenogroup labels independently derived by latent class analysis (LCA) in the GSTT external replication cohort Abbreviations: CKM, Cardiovascular-Kidney-Metabolic; Elderly-AD, Elderly-Atrial Dysfunction; LCA, Latent Class Analysis.*

**Supplemental Table S9: Five-Year Phenogroup Transition Matrix**

Percentages represent the proportion of patients in each baseline phenogroup who transitioned to each destination state by Year 5. Death column includes all deaths occurring during 5-year follow-up, including those after phenogroup transition.

| **Baseline Phenogroup** | **Remained Stable** | **To Cardiovascular-Kidney-Metabolic (%)** | **To Elderly–Atrial Dysfunction (%)** | **To Obesity–Predominant (%)** | **To Young–Low Comorbidity (%)** | **Death** |
| --- | --- | --- | --- | --- | --- | --- |
| **Young-Low Comorbidity** | 35.7% | 11.8% | 13.8% | 11.2% | — | 27.5% |
| **Obesity-Predominant** | 43.5% | 25.7% | 5.2% | — | 0.0% | 25.5% |
| **Elderly-Atrial Dysfunction** | 47.4% | 7.6% | — | 0.1% | 0.0% | 45.0% |
| **Cardiovascular-Kidney-Metabolic** | 51.8% | — | 2.6% | 0.0% | 0.0% | 45.6% |

**Supplemental Table S10: Mortality Patterns by Transition Status.** Distribution of deaths by whether patients remained in their baseline phenogroup or transitioned to other phenogroups

| Baseline Phenogroup | Total Deaths (N) | Total Mortality (%) | Deaths While Stable (N) | Deaths After Transition (N) | % Deaths While Stable |
| --- | --- | --- | --- | --- | --- |
| Young-Low Comorbidity | 133 | 27.5% | 2 | 131 | 1.5% |
| Obesity-Predominant | 122 | 25.5% | 9 | 113 | 7.4% |
| Elderly-Atrial Dysfunction | 357 | 45.0% | 335 | 22 | 93.8% |
| Cardiovascular-Kidney-Metabolic | 213 | 45.6% | 200 | 13 | 93.9% |
| Overall | 825 | 37.1% | 546 | 279 | 66.2% |

**Supplementary Table S11.** Baseline characteristics of patients with the Young-Low Comorbidity phenotype according to subsequent phenogroup transition

| **Characteristic** | **Remained YLC (n=256)** | **Transitioned (n=231)** | **SMD** |
| --- | --- | --- | --- |
| Age | 52.00 [39.75, 65.00] | 67.00 [54.50, 75.00] | **0.81** |
| H_2_FPEF score | 2 [1, 3] | 3 [2, 5] | **1.01** |
| E/e′ average | 8.20 [6.80, 10.25] | 10.89 [8.62, 13.59] | **0.67** |
| Septal E/e′ | 9.48 [7.32, 11.81] | 12.31 [9.85, 15.78] | **0.65** |
| IVSd (cm) | 1.00 [0.86, 1.14] | 1.14 [0.99, 1.33] | **0.64** |
| LV mass index (g/m^2^) | 82.00 [70.70, 94.92] | 94.86 [81.33, 109.18] | **0.58** |
| LA volume index (cm^2^/m^2^) | 27.77 [23.94, 32.00] | 32.43 [27.90, 41.63] | **0.56** |
| TR velocity (m/s) | 2.52 [2.25, 2.96] | 2.73 [2.50, 3.04] | 0.34 |
| Creatinine (umol) | 69.50 [55.75, 84.00] | 81.00 [64.00, 115.00] | 0.46 |
| BMI (kg/m^2^) | 27.70 [24.79, 31.59] | 30.28 [26.30, 35.10] | 0.38 |
| NTproBNP umol/l | 1094 [653, 1704] | 1502 [887, 2622] | 0.26 |
| AF | 4 (1.4) | 10 (4.3) | 0.18 |
| Diabetes | 1 (0.5) | 6 (2.4) | 0.16 |
| Hypertension | 6 (2.3) | 7 (3.1) | 0.053 |
| CAD | 7 (2.7) | 6 (2.7) | 0.001 |
| CKD | 24 (9.5) | 24 (10.6) | 0.035 |

***SMD, standardised mean difference. SMDs values ≥0.5 were considered indicative of moderate and ≥0.8 of large differences. Data are presented as median [IQR] or n (%). Abbreviations:*** *TR, tricuspid regurgitation; IVSd, interventricular septal thickness end diastole; BMI, body mass index; AF, atrial fibrillation; CAD, coronary artery disease; CKD, chronic kidney disease.*

**Supplemental Table S12.** Survival estimates at 1, 2 and 5 years stratified by phenogroups. Kaplan-Meier survival estimates with upper and lower 95% Confidence Intervals

| **Year** | **Elderly-Atrial Dysfunction,**  N = 703 | **Cardiovascular-Kidney-Metabolic**,  N = 503 | **Obesity predominant**,  N = 530 | **Young Low Comorbidity**,  N = 487 |
| --- | --- | --- | --- | --- |
| **Year 1** | 76.1 (73.2-79.5) | 79.7 (76.3-83.3) | 92.5 (90.2-94.7) | 87.1 (84.1-90.1) |
| **Year 2** | 68.4 (65.1-71.9) | 71.2 (67.3-75.2) | 87.4 (84.6-90.2) | 84.0 (80.8-87.3) |
| **Year 5** | 54.3 (50.0-58.4) | 54.8 (50.3-59.7) | 76.7 (73.0-80.6) | 74.8 (70.8-79.0) |

**Supplemental Figure S1.** **Cohort selection flow diagram**. Patients were identified from the EHRs of over 1,000,000 individuals using NLP to detect HF mentions and echocardiographic evidence of preserved left ventricular ejection fraction (LVEF ≥ 50%) (n = 3,727). Exclusion criteria included lack of objective evidence of diastolic dysfunction or raised left ventricular (LV) filling pressures (n = 347), echocardiography more than one year from first HF mention (n = 899), and the presence of significant valvular disease, cardiomyopathy, or amyloidosis (n = 258). HFpEF was defined using two parallel criteria: (1) clinician-documented HFpEF (n = 231) and (2) meeting ESC guideline-based HFpEF diagnostic criteria (n = 1,992). The final study cohort (n = 2,223) comprised patients who fulfilled either or both definitions.

**Supplemental Figure S2. Kaplan-Meier survival curves for all-cause mortality across HFpEF phenogroups in the external replication cohort (GSTT, N = 3,349).** Log-rank tests were used to assess significant differences in survival across clusters. 5-year survival estimates were: Young-Low Comorbidity = 63% (95% CI, 59-67%), Elderly-Atrial Dysfunction = 37% (95% CI, 33-42%), Cardiovascular-Kidney-Metabolic = 51% (95% CI, 47-54%) and Obesity-Predominant = 56% (95% CI, 51-62%). Cox proportional hazards regression confirmed the Elderly-Atrial Dysfunction group had the highest risk of mortality compared to the Young-Low Comorbidity reference group (HR 2.07, 95% CI 1.76–2.44, p < 0.001), followed by the Cardiovascular-Kidney-Metabolic group (HR 1.46, 95% CI 1.27–1.69, p < 0.001) and the Obesity-Predominant group (HR 1.20, 95% CI 1.02–1.48, p = 0.03).

**Supplemental Figure S3.** **Variable feature importance**. Feature importance in the Random Forest model used for phenogroup reassignment at follow up. Bar plots compare variable importance using Mean Decrease in Gini. Variables are ranked in ascending order of importance, with those with the greatest Mean Decrease in Gini having the most importance.

**Supplemental Figure S4.** Baseline predictors of transition from the Young-Low Comorbidity phenogroup.

*Odds ratios (95% CI) from a multivariable logistic regression model estimating the association between baseline clinical characteristics and subsequent transition from the Young-Low Comorbidity phenogroup. Odds ratios for continuous variables are expressed per standard deviation increase*

**Supplemental References**

1.	Jackson R, Kartoglu I, Stringer C, Gorrell G, Roberts A, Song X, Wu H, Agrawal A, Lui K, Groza T, Lewsley D, Northwood D, Folarin A, Stewart R, Dobson R. CogStack - Experiences of deploying integrated information retrieval and extraction services in a large National Health Service Foundation Trust hospital. *BMC Medical Informatics and Decision Making*. 2018;18(1):1–13.

2.	Kraljevic Z, Searle T, Shek A, Roguski L, Noor K, Bean D, Mascio A, Zhu L, Folarin AA, Roberts A, Bendayan R, Richardson MP, Stewart R, Shah AD, Wong WK, et al. Multi-domain clinical natural language processing with MedCAT: The Medical Concept Annotation Toolkit. *Artificial Intelligence in Medicine*. 2021;117:102083.

3.	Farajidavar N, O’Gallagher K, Bean D, Nabeebaccus A, Zakeri R, Bromage D, Kraljevic Z, Teo JTH, Dobson RJ, Shah AM. Diagnostic signature for heart failure with preserved ejection fraction (HFpEF): a machine learning approach using multi-modality electronic health record data. *BMC Cardiovascular Disorders*. 2022;22(1):567.

4.	Bean DM, Kraljevic Z, Searle T, Bendayan R, Kevin O, Pickles A, Folarin A, Roguski L, Noor K, Shek A, Zakeri R, Shah AM, Teo JTH, Dobson RJB. Angiotensin‐converting enzyme inhibitors and angiotensin II receptor blockers are not associated with severe COVID‐19 infection in a multi‐site UK acute hospital trust. *European Journal of Heart Failure*. 2020;22(6):967–974.

5.	Searle T, Kraljevic Z, Bendayan R, Bean D, Dobson R. MedCATTrainer: A Biomedical Free Text Annotation Interface with Active Learning and Research Use Case Specific Customisation. In: *Proceedings of the 2019 Conference on Empirical Methods in Natural Language Processing and the 9th International Joint Conference on Natural Language Processing (EMNLP-IJCNLP): System Demonstrations*. Stroudsburg, PA, USA: Association for Computational Linguistics; 2019:139–144.
